# Adherence to the DASH Diet in the Spanish Population and Its Environmental Impact: An Ecological Study

**DOI:** 10.3390/nu18111822

**Published:** 2026-06-05

**Authors:** Sergio Rodríguez Núñez, Laura Álvarez-Álvarez, Vicente Martín-Sánchez, Lucia Callejo Quintanilla, Isabel García-Cuesta, Beatriz San-Miguel, Antonio J. Molina

**Affiliations:** 1 Group of Investigation in Interactions Gene-Environment and Health (GIIGAS), Universidad de León, 24007 León, Spain; srodrn@unileon.es (S.R.N.); vicente.martin@unileon.es (V.M.-S.); lcalq@unileon.es (L.C.Q.); ajmolt@unileon.es (A.J.M.); 2 Institute of Biomedicine (IBIOMED), Universidad de León, 24007 León, Spain; bsanv@unileon.es; 3 Consortium for Biomedical Research in Epidemiology & Public Health (CIBER Epidemiología y Salud Pública-CIBERESP), 28029 Madrid, Spain; igarcc@unileon.es; 4 Instituto Universitario de Oncología del Principado de Asturias (IUOPA), Department of Medicine, University of Oviedo, 33006 Oviedo, Asturias, Spain

**Keywords:** dietary patterns, environmental impact, ecological footprint, life cycle assessment, cardiovascular health, DASH, sustainability

## Abstract

Background/Objectives: Plant-based dietary patterns like the Dietary Approaches to Stop Hypertension (DASH) reduce cardiovascular risk, which is a leading cause of mortality globally and in Spain. Diet is also a major environmental determinant, highlighting the need to evaluate public health alongside environmental sustainability. The aim of this study was to analyze the evolution of adherence to the DASH dietary pattern in Spain between 2006 and 2023 and evaluate its relationship with environmental sustainability indicators. Methods: This was an ecological epidemiological study. Food consumption data were harmonized into daily servings to calculate annual DASH scores using a standard 80-point methodology. Environmental impact was assessed by calculating the comprehensive Ecological Footprint (EF) using the Agribalyse^®^ 3.2 database. The study utilized open data from the Spanish Household Budget Surveys, capturing the consumption habits of approximately 24,000 randomly selected Spanish households annually from 2006 to 2023. The primary measures evaluated were the annual DASH adherence index score and the overall environmental Ecological Footprint. Temporal trends were evaluated using segmented regression models selected via the Akaike Information Criterion and Davies test. Pareto analysis determined individual food group environmental contributions, and correlations assessed the relationship between DASH scores and the EF. Results: DASH adherence increased by 8.26% over the study period, peaking in 2020. The EF demonstrated an overall decrease over time, largely driven by reduced consumption of meat, fish, and eggs. A strong inverse correlation was found between the DASH score and the EF (r = −0.8237 (95% CI: −0.932 to −0.580; *p* < 0.001)). Conclusions: A shift toward the DASH dietary pattern in Spain demonstrates potentially convergent health and environmental associations, promoting population cardiovascular health potential while simultaneously mitigating environmental impacts.

## 1. Introduction

Cardiovascular diseases (CVD) remain the leading cause of death worldwide, accounting for 20.5 million deaths in 2021, approximately one third of all global deaths [1]. In Spain in 2023, CVD is also the primary cause of mortality (26%), surpassing cancer (24.8%) and respiratory diseases (19.3%) [2].

Cardiovascular risk is a result of both non-modifiable factors, such as age, sex, and genetic load [3,4,5], and modifiable factors of behavioral or pathophysiological origin, including smoking, a sedentary lifestyle, dyslipidemia, hypertension and diet [6,7,8]. Among these, hypertension is one of the most prevalent, affecting more than 80% of the European adult population over 60 years old [9].

Scientific evidence supports that non-pharmacological interventions, particularly dietary changes, are essential for both preventing CVD and enhancing the effectiveness of pharmacological treatments [10,11]. In this context, plant-based dietary patterns, such as the Dietary Approaches to Stop Hypertension (DASH), have been shown to reduce blood pressure, overall cardiovascular risk, and metabolic comorbidities [12,13]. The DASH pattern emphasizes a high intake of fruits, vegetables, legumes, whole grains and low-fat dairy products, alongside a reduced consumption of meat, fats and sweets.

Beyond its impact on health, diet is a major environmental determinant. It is estimated that one-third of global greenhouse gas emissions originate from food systems, especially from animal products [14,15]. To evaluate this impact different databases are used to quantify environmental impact indicators in a standardized way. Examples of these databases are Agribalyse, EAT-Lancet or SU-EATABLE LIFE [16,17,18].

This is consistent with the “diet–health–environment trilemma”, which highlights the need to balance human and planetary health [19]. This concept has given rise to the development of the planetary diet, a dietary regime which involves a reduction in the consumption of animal products, with these products being replaced by plant-based alternatives. The aim of this dietary regime is twofold: firstly, to improve human health, and secondly, to achieve environmental sustainability [20]. As demonstrated by Muszalska et al. [21], this approach has been shown to be effective in the prevention of obesity and chronic diseases.

In Europe, a trend towards more Westernized diets and greater consumption of ultra-processed foods could compromise both public health and environmental sustainability [22,23]. Therefore, a simultaneous assessment of adherence to healthy dietary patterns and their environmental impact can have importance.

Ecological studies based on population-level data are particularly useful for examining long-term temporal trends and generating hypotheses regarding associations between dietary patterns and health or environmental indicators at the population level [24]. In the present context, the use of nationally aggregated dietary data enables the assessment of temporal changes in DASH adherence and their relationship with ecological footprint indicators in Spain.

The objective of this study was to analyze the evolution of adherence to the DASH dietary pattern in Spain and its relationship with environmental sustainability indicators, using national food consumption data.

## 2. Materials and Methods

This ecological epidemiological study assesses adherence to the DASH diet in the Spanish population and its associated environmental impact between 2006 and 2023.

Food consumption data were sourced from the Household Budget Surveys (HBS), which are openly available from the Spanish National Statistics Institute (INE) [25]. The survey is conducted on an annual basis and collects socio-economic information on the standard of living and consumption of approximately 24,000 Spanish households. These households are randomly selected and participate in the survey for two consecutive weeks each year for two consecutive years. These households are tasked with the collection of information pertaining to their expenditure over a two-week period, utilizing questionnaires and interviews as data collection instruments.

The classification employed for the analysis of data from 2006 to 2015 was Classification of Individual Consumption according to Purpose (COICOP). From 2016 onwards, the European Classification of Individual Consumption by Purpose (ECOICOP) classification was utilized, with the objective of facilitating comparisons with other statistics, such as the Consumer Price Index.

The DASH dietary score was operationalized using household food consumption data from the Spanish Household Budget Survey (EPF). Given that EPF does not provide nutrient composition data, the score was constructed based on food group proxies rather than nutrient-level components (e.g., sodium, potassium, magnesium, calcium). Therefore, the index should be interpreted as a DASH-like dietary pattern indicator reflecting food group availability at the population level, rather than a full nutrient-based DASH adherence score.

The DASH diet originated from a clinical study in the United States that examined the effects of different eating patterns on blood pressure [12]. It was subsequently adopted by the U.S. government as a set of general dietary recommendations for the population [26]. This dietary pattern is based on 10 food groups with high consumption of vegetables, legumes, fruits, low-fat dairy, and high-fiber cereals, along with low intake of meats, fats, and sweets [27].

For the evaluation of the DASH index [12], we used the scoring methodology outlined by Günther et al. [28]. This method establishes minimum and maximum scores for each food group, with a maximum score of 80 points. For intermediate consumption levels, the score was calculated using a linear weighting between the established minimum and maximum serving sizes, as detailed in Table 1. The final DASH index score is the sum of the scores obtained from the 10 food groups, calculated independently for each study year.

No missing data were identified in the HBS datasets; therefore, no imputation procedures were required. The HBS data were first separated by year for independent evaluation. The data were then converted to daily food consumption in servings. Serving size definitions were adapted from the “PREvención con Dieta MEDiterránea” (PREDIMED) study [29], due to their prior use and cultural applicability in Spanish dietary assessment. Although originally developed within the context of Mediterranean diet research, these portion definitions were used solely as standardized operational criteria to classify food consumption from EPF data and do not imply conceptual equivalence between Mediterranean and DASH dietary patterns. This approach facilitated comparability and interpretation within the Spanish dietary context.

The foods were classified according to HBS codes and harmonized with the 10 food groups required for the DASH assessment. In some cases, several third-level HBS codes were combined into a single DASH group, while in others, fourth-level subcategories were used to refine the classification; full correspondence tables are provided in Supplementary Material S1. Once the groups were established, the servings of all foods within each group were summed to determine the total servings for each category.

Specifically, to classify high-fiber cereals, we used data from the annual INE food consumption panel [30]. We calculated the percentage of whole-meal bread and high-fiber cereals relative to total per capita consumption for all cereals and breads. For low-fat dairy products, we considered semi-skimmed and skimmed milk, as these categories are explicitly available in the HBS data.

The environmental impact analysis was conducted using the French database Agribalyse^®^ 3.2, which is the most current version available [16], specifically utilizing its environmental footprint section.

We used a comprehensive index provided by Agribalyse, the Ecological Footprint (EF), which combines the 19 indicators available in their data into an overall environmental impact score. This EF is calculated according to European standards by normalizing the score for each indicator by its European average and then computing a weighted score.

For the environmental analysis, we calculated the daily consumption of each food item in grams. The food items were then grouped based on their basic, raw form, excluding prepared dishes. All specific choices made for this classification are listed in an attached Excel file in Supplementary Material S2, along with a table of initial examples. When an HBS entry corresponded to more than one Agribalyse food item, an average of the environmental impact scores was calculated.

Temporal trends in DASH scores and environmental indicators were first described using line graphs. A segmented regression analysis was performed to assess potential changes in temporal trends. Three candidate models were evaluated: a model with no change points (linear trend), a model with one change point, and a model with two change points. We used the segmented package in R [31] and the Akaike Information Criterion (AIC) to select the model that best fit our data, and the Davies test [32] was evaluated to determine if there would be a change in tendency. The test is necessary because standard hypothesis testing fails when a parameter (in this case, the breakpoint or “change-point”) exists only under the alternative hypothesis but not under the null hypothesis (which assumes a straight line).

To easily visualize the environmental impact contributed by each food group, a Pareto analysis was performed. This analysis involved creating graphs that represent the relative and cumulative frequencies of each food group’s contribution to each environmental impact measure [33].

Pearson correlation coefficients were calculated to assess the association between DASH adherence and ecological footprint. To evaluate potential residual autocorrelation associated with the temporal structure of the data, the Durbin–Watson test was applied to the regression residuals of ecological footprint on DASH adherence. Spearman’s rank correlation coefficient (ρ) was additionally computed as a non-parametric sensitivity analysis. Correlation coefficients are presented with corresponding 95% confidence intervals calculated using Fisher’s z-transformation.

Given the methodological transition from COICOP to ECOICOP classification in 2016, sensitivity analyses were performed by (i) excluding transition years (2015–2016), and (ii) analysing pre-ECOICOP (2006–2015) and post-ECOICOP (2016–2023) periods separately. Structural discontinuity at the transition point was evaluated using a Chow test.

Supplementary analyses using individual environmental indicators, including climate change emissions, land use, and water resource depletion, showed patterns broadly consistent with the composite ecological footprint index (Supplementary Material S3).

Excel version 2025 (Microsoft Corporation, Redmond, WA, USA), Python version 3.13.3 (Python Software Foundation), R version 4.5.0 (R Foundation for Statistical Computing, Vienna, Austria), and STATA version 14.2 (Stata corporation, College Station, TX, USA) tools were used for data processing, statistics, and graph creation.

## 3. Results

**Figure 1:**
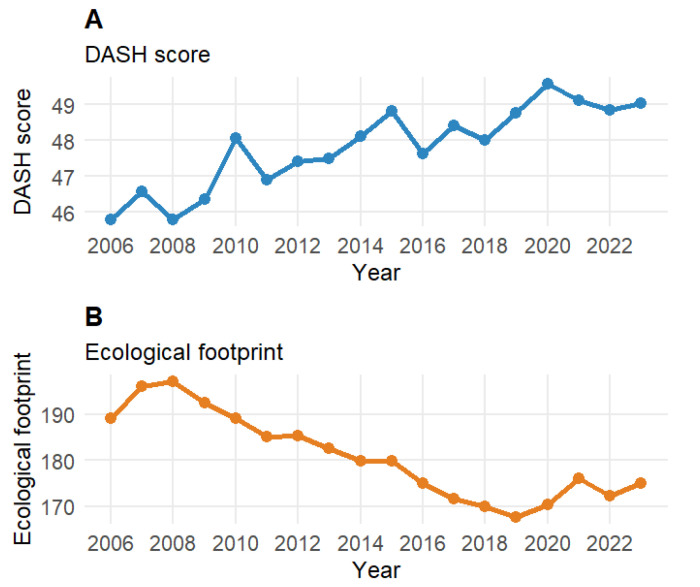
Temporal trends in DASH adherence and ecological footprint in Spain (2006–2023). (**A**) Annual mean DASH score estimated from Spanish Household Budget Survey (EPF) data. (**B**) Annual ecological footprint associated with household food consumption over the same period. Data are presented as independent annual cross-sectional estimates.

Adherence to the DASH index increased over the study period, with scores consistently above half of the maximum possible value in all years and reaching a peak in 2020. An overall increase of 8.26% was observed during the study period (Figure 1); a table is also present in the Supplementary Material S4.

An overall decrease in environmental pressure was observed, as indicated by the EF score showing its tendency in Figure 1. The trends for every environmental indicator can be appreciated in Supplementary Material S5.

**Figure 2:**
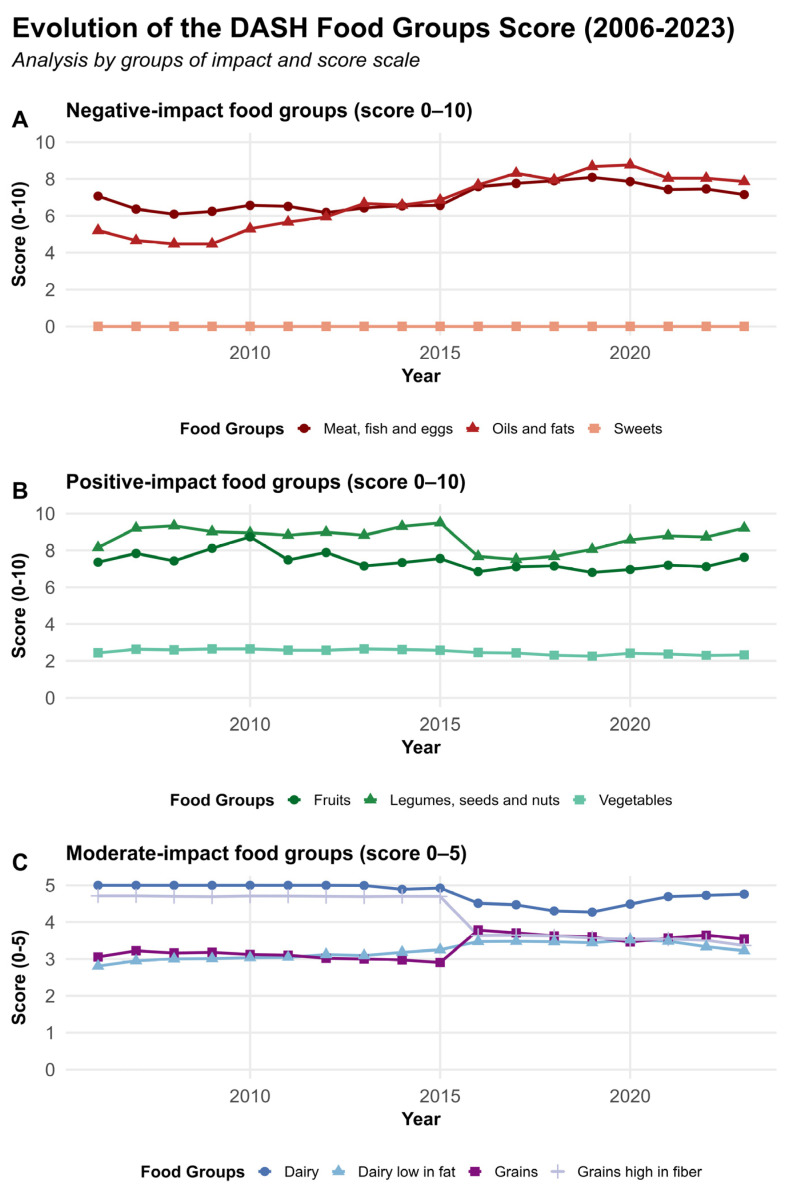
Evolution of food groups during the study period divided in groups: (**A**)—Food groups out of 10 points for DASH score, where less consumption produces more DASH score; (**B**)—Food groups out of 10 points for DASH score, where more consumption produces more DASH score; (**C**)—Food groups out of 5 points for DASH score.

Figure 2 illustrates the temporal contribution of each food group to the total DASH score. While the ‘oils and fats’ group exhibited the largest relative increase over time, the categories making the greatest overall contributions throughout the period were fruits; oils and fats; and meat, fish, and eggs. Conversely, vegetables and sweets contributed the least to the total score. Notably, the significant fluctuations observed between 2015 and 2016 are likely attributable to revisions in the survey methodology.

The temporal trend analysis results indicated that the EF had a very high adherence to a segmented model (AIC = 77.810; R2 = 0.975; R2 Adjusted = 0.965; Davies *p* value = 0.005). This model identified breakpoints approximately in 2007 and 2019. The trend was characterized by an initial increase in impact, followed by a decrease during the next period, and a subsequent renewed increase after 2019. Regarding DASH adherence, the analysis revealed an ascending linear regression which also exhibited a high goodness-of-fit (AIC = 32.170; R2 = 0.801; R2 Adjusted = 0.789; Davies *p* value = 0.483). Graphs detailing each environmental impact and DASH trends are available in Supplementary Material S6.

The Pareto analysis reveals that the fish, meat, and eggs food group is the primary contributor to environmental impact, accounting for more than 50% of the impact. This information is summarized in the Pareto charts in Supplementary Material S7 for every environmental indicator.

Pearson correlation demonstrated a strong inverse association between DASH adherence and ecological footprint (r = −0.8237, 95% CI: −0.932 to −0.580; *p* < 0.001). Similar results were obtained using Spearman’s correlation (ρ = −0.7379, 95% CI: −0.896 to −0.413; *p* < 0.001).

Sensitivity analyses were conducted to evaluate the potential impact of the 2015–2016 transition from COICOP to ECOICOP classification.

The year-to-year changes observed at the transition point were modest and followed the overall temporal direction of the series (DASH: −2.4%; ecological footprint: −2.7%). A Chow test detected no statistically significant structural break in the DASH–ecological footprint relationship in 2016 (F = 1.58, *p* = 0.24).

Correlation analyses remained robust when excluding transition years (r = −0.854, 95% CI: −0.948 to −0.622; *p* < 0.001) and during the pre-ECOICOP period (2006–2015: r = −0.773, 95% CI: −0.944 to −0.280; *p* = 0.009). No statistically significant association was observed in the post-ECOICOP period alone (2016–2023: r = −0.049; *p* = 0.908). Detailed results are presented in Supplementary Material S8.

## 4. Discussion

The primary objective of this study was to analyze the evolution of adherence to the DASH diet in Spain from 2006 to 2023 and its environmental impact, by analyzing the temporal trends and the correlations between these two concepts.

The findings of the present study demonstrate a progressive approximation to the DASH diet recommendations in Spanish consumption data from 2006 to 2023. This trend is typified by an initial decline in the early years, followed by a rapid increase, and a less pronounced increase in the later years. This tendency is replicated in other articles, for example, in Israel between 2005 and 2015 [34]. In addition, the bibliography suggests that whilst diagnosis may foster a modest improvement in dietary habits, achieving optimal adherence to the DASH diet remains a significant challenge for the Mexican population. For instance, in 2012, the mean DASH diet adherence score for men with undiagnosed hypertension in Mexico was a mere 30.7 out of 80, whilst the score for those with a confirmed diagnosis was marginally higher at 33.5. Furthermore, only 34.7% of men with diagnosed hypertension met the adherence criteria, compared with 21.8% of the undiagnosed group [35].

However, these comparisons should be interpreted with caution, as differences in dietary assessment methods, healthcare systems, socioeconomic structures, and temporal coverage limit direct comparability between countries. Therefore, these observations are intended as contextual benchmarks rather than direct quantitative equivalences.

The findings of this study indicate an increase maintained over time in adherence to the DASH diet among all the years of study in the Spanish population. The variation in the DASH score was approximately 5 points between the minimum and maximum values. It may be important to mention the hypothesis that a five-point change may yield positive clinical outcomes in individuals that has been proposed in a number of studies on various diseases, including hypertension [36], cardiovascular risk factors [37,38], inflammation [39] and even a potential reduction in all-cause mortality has been proposed with a five-point change in adherence to the DASH diet [40].

There are studies that show that greater adherence to the DASH diet lowers the risk of developing hypertension and regarding our results, we can conclude that the risk of developing this condition should be reduced [41,42].

Given the increased adherence to the DASH diet observed in Spanish consumption data, alongside the previously cited literature, it is plausible to suggest that the observed dietary trends may be consistent with population-level patterns associated with lower cardiovascular risk, although causal inference cannot be established from this ecological design. This hypothesis is supported by the downward trend in age-adjusted cardiovascular mortality in Spain. Notably, provisional data from 2024 indicate that, for the first time in history, tumors have surpassed cardiovascular disease as the leading cause of death [43]. These findings stand in contrast to recent predictions by the American Heart Association, which projected an increase in diet-related risk across Western populations [44].

The environmental impact results were like those of the DASH analysis, showing a sustained improvement over time in most cases. A change in this trend was observed around 2007 and again in 2019. These periods correspond to significant economic, social, and political instability, coupled with high inflation, which may be driving these shifts, which might be linked somehow. This idea is supported by previous studies showing a significant decline in household vegetable consumption during and after the 2008 recession [45]. This effect was particularly pronounced among disadvantaged or food-insecure households [46].

Studies have shown a clear reduction in overall dietary quality in high-income countries, with a specific decrease in fruit and vegetable consumption. In contrast, the impact was less consistent in middle-income countries [45]. For example, in the United States, food-insecure households reported a lower intake of vegetables, particularly dark green varieties, during the recession [47]. Similar trends were observed in European countries such as Italy [48] and Spain [46], where the likelihood of adopting healthy behaviors, such as the regular consumption of vegetables, decreased. These effects were particularly evident in regions and groups most affected by the economic recession.

The COVID-19 pandemic also prompted studies on potential changes in dietary patterns. Some research has shown an increase in calorie consumption [49], driven by a rise in the intake of sugars and snacks, producing a shift in diet that may have contributed to increased rates of obesity and overweight [50].

The Pareto principle [51] states that a small number of variables can account for roughly 80% of the total effect, with the remaining variables having a negligible impact. The results of this study are consistent with this principle, as animal products alone contribute a percentage close to this value regarding their overall environmental impact.

Studying this statement, the overall improvement in environmental impact can, therefore, be explained by the reduction in the consumption of meat, fish, and eggs, as these food groups are the main contributors to most environmental categories. This may provide a basis for the formulation of new and effective policies. It is evident that the most significant environmental impact is attributable to a limited number of food groups: meat, fish and eggs are of primary importance, and fruit is second in line.

The robustness of the observed association was further supported by sensitivity analyses and by the absence of statistically significant residual autocorrelation, although the ecological and longitudinal nature of the data requires cautious interpretation.

These findings are consistent with previous research that has found a strong link between dietary choices and both environmental impact [52,53] and human health [18,54]. The data suggests that improving both human and planetary health is achievable through a reduction in the consumption of animal products [55].

Furthermore, there has been proposed the importance of promoting healthy and environmentally sustainable diets from a public health perspective by a randomized clinical trial studying a nutritional intervention with the energy-reduced Mediterranean Diet and the environmental impact of the diet [56], while these findings are not directly comparable to ecological associations, they provide complementary evidence supporting the potential health relevance of dietary patterns characterized by high plant-based food intake and reduced consumption of animal products.

In Spain, both plant-based and Mediterranean diets have been studied as tools for achieving human and planetary health [57,58]. A recent study analyzing the same HBS data found that adherence to the Mediterranean diet remained virtually unchanged over time with a change of 2.94% of increase between its maximum and minimum [59], which contrasts with our findings for the DASH diet with an increase of 8.26%.

This discrepancy may be due to the differences in how the two diets are assessed. The Panagiotakos index [60], used to evaluate the Mediterranean diet, only considers red meat, whereas our DASH assessment includes total meat consumption. Furthermore, it focuses on whole grains rather than all grains and only accounts for olive oil as a beneficial fat, unlike our approach which considers all fats. Lastly, the Panagiotakos index includes alcohol consumption, which is not a factor in the DASH diet. Regarding the environmental impact of the diet, we found a similar tendency where the impact has been reduced, even with a different methodology and parameters [18].

Our study, which analyzed the relationship between the DASH diet and environmental sustainability, yielded positive results consistent with previous research [61,62]. These findings support the concept of the diet, environment, and health trilemma [19,63], demonstrating that the most effective way to address these challenges is through an integrated approach.

Our findings suggest that it is possible to align the population’s cardiovascular health alongside changes in dietary patterns with the health of the planet, although these associations should not be interpreted as causal. This crucial connection should be considered when developing new policies aimed at halting the recent decline in dietary quality.

Our findings may be interpreted within the framework of the “diet–health–environment trilemma”, which emphasizes the need to balance human and planetary health [19]. In this context, dietary models aligned with the planetary diet, characterized by lower consumption of animal-source foods and greater reliance on plant-based alternatives, have been proposed as strategies to simultaneously improve health outcomes and reduce environmental impact [20]. Although the DASH diet was originally designed to prevent and manage hypertension, several of its core characteristics—including higher consumption of fruits, vegetables, and plant-based foods and lower intake of certain animal-derived products—overlap with principles of sustainable dietary patterns. Previous evidence suggests that such approaches may contribute to the prevention of obesity and chronic diseases [21]. Therefore, the associations observed in the present study between DASH adherence and ecological footprint indicators may be interpreted within this broader diet–health–environment framework, while acknowledging the ecological nature of the study design.

This study has several methodological limitations that should be considered. First, it was conducted using population-level data rather than individual-level data. Another key limitation is the quality and reliability of the environmental indicators, many of which are classified as improvable by their own authors.

An additional limitation inherent to ecological studies is the potential for ecological fallacy. Because analyses are based on aggregated household- and population-level data, observed associations cannot be directly extrapolated to individual behaviors or outcomes. For example, an increase in population-level DASH adherence occurring alongside reductions in ecological footprint or cardiovascular mortality does not necessarily imply that individuals with higher DASH adherence experienced lower environmental impact or cardiovascular risk. Consequently, the findings should be interpreted as ecological associations reflecting population-level temporal patterns rather than individual-level relationships.

Another limitation relates to the inability to control for potentially relevant confounding factors inherent to ecological analyses based on secondary aggregated data. The present study did not adjust for demographic changes (including population ageing, migration, or household composition), socioeconomic conditions (such as income distribution, unemployment, or food accessibility), changes in healthcare and pharmacological management, increasing health awareness, or variations in physical activity patterns over time. These factors may have influenced dietary behaviors, cardiovascular indicators, and environmental trends during the study period and therefore could partially contribute to the observed associations. Consequently, the findings should be interpreted as population-level temporal associations rather than independent effects attributable solely to dietary patterns.

Despite the ecological nature of our study being a limitation, it is important to highlight the usefulness of this type of study in paving the way for future research that will generate new knowledge and help lay the foundations for new policies that will guide the future. Also, it would be of great use to replicate this methodology in other countries to find the situation in other places and have comparability with other regions. Changes in HBS classification and methodology, particularly the transition from COICOP to ECOICOP around 2015–2016, may also have introduced discontinuities in the time series, as reflected in some of the observed fluctuations.

Although each annual EPF wave is based on an independent cross-sectional sample of households, analyses based on aggregated annual time series may be affected by temporal autocorrelation. Therefore, correlation results should be interpreted as exploratory associations rather than inferential causal relationships.

An additional limitation of this study is the use of household-level data from the EPF, which does not capture individual dietary intake within households. This implies that within-household variability (e.g., differences by age, sex, or individual dietary preferences) cannot be assessed. Consequently, the DASH-like indicator reflects average household consumption patterns and may not represent individual dietary behavior. Moreover, potential differences in household composition (e.g., size, age structure, urban–rural distribution, and socioeconomic status) may influence food acquisition patterns and introduce residual confounding that cannot be fully controlled in this ecological design.

The ecological footprint metric used in this study also presents limitations that should be acknowledged. Environmental impact estimates were derived from the Agribalyse^®^ database, originally developed using French agricultural and food production systems. Although widely applied in life-cycle assessment studies, differences in production methods, supply chains, and agricultural practices between France and Spain may limit the direct transferability of some environmental estimates.

Additionally, the ecological footprint represents a composite environmental indicator integrating multiple life-cycle dimensions into a single score. While this facilitates overall interpretation and comparison, it may obscure the relative contribution and weighting of individual environmental components.

To address this limitation, supplementary analyses were performed using selected individual environmental indicators, including greenhouse gas emissions, water use, and land use. These complementary analyses yielded patterns broadly consistent with the overall ecological footprint results, supporting the robustness of the observed associations while acknowledging that no single environmental metric fully captures dietary sustainability.

Despite these limitations, our study has several key advantages. We used a French environmental database, Agribalyse^®^ 3.2, which offers geographical, cultural, and social proximity to our Spanish study population. This provides context-specific data that reflects local conditions, such as climate, land use, and community behaviors [64], making it more representative than other databases. Furthermore, a significant strength of this research is its ability to generate knowledge from existing information at a reasonable cost, allowing for the establishment of correlations and hypotheses that can be tested in subsequent studies.

## 5. Conclusions

The findings of this study indicate a consistent rise in DASH scores within the Spanish population from 2006 to 2023, observations that suggest a potential for these results to contribute to a reduction in the risk of hypertension and other cardiovascular diseases.

Accompanying this rise in DASH adherence, we observed a general downward trend in the main ecological footprint indicators. This transition toward a diet richer in plant-based foods and lower in animal products demonstrates a dual benefit: it promotes human health while also mitigating the environmental impact. The results underscore that public health and environmental sustainability are not mutually exclusive but can be effectively addressed through shared strategies.

## Figures and Tables

**Table 1:** Summary table of the DASH index.

Food Group	Score	Score Frequency	Servings for Maximum Score	Servings for Minimum Score
Vegetables	0–10	Day	≥4	0
Legumes, seeds and nuts	0–10	Week	≥4	0
Fruit	0–10	Day	≥4	0
Grains—Total	0–5	Day	≥6	0
Grains—High in fiber	0–5	Day	≥50% ^1^	0
Meat, fish and eggs	0–10	Day	≤2	≥4
Dairy products—Total	0–5	Day	≥2	0
Dairy products—Low in fat	0–5	Day	≥75%	0
Oils and fats	0–10	Day	≤3	≥6
Sweets	0–10	Week	≤5	≥10

Source: Adaptation from the table shown in Günther et al. [28]. Note: ^1^: Percentage of high-in-fiber grains in the total grain consumption, more information about the food groups selection is available in Supplementary Material S1.

## Data Availability

Data available in the Spanish National Statistical Institute on its website.

## Supplementary Materials

The following supporting information can be downloaded at https://www.mdpi.com/article/10.3390/nu18111822/s1. Supplementary Material S1: Food groups correspondence into food categories; Supplementary Material S2: Food items correspondence choice from Agribalyse data set into the household budgets survey items; Supplementary Material S3: Pearson and Spearman correlations between DASH adherence and selected environmental indicators derived from Agribalyse^®^ life-cycle assessment data (2006–2023). Confidence intervals for Pearson correlations were estimated using Fisher’s z-transformation; Supplementary Material S4: DASH and Ecological footprint values exposed in the Figure 1; Supple-mentary Material S5: Environmental impact indicators trends; Supplementary Material S6: Graphs detailing each environmental impact and DASH trends; Supplementary Material S7: Pareto charts for all the environmental impact indexes; Supplementary Material S8: Sensitivity analyses of the association between DASH adherence and ecological footprint across study periods.
