# Supplementary material for: Adherence to the DASH Diet in the Spanish Population and Its Environmental Impact: An Ecological Study
Source: Nutrients. 2026 Jun 5;18(11):1822. doi: 10.3390/nu18111822 (PMC13259029; doi:10.3390/nu18111822)
Supplement: Supplementary file 1 [file nutrients-18-01822-s001.zip › SM5-7.pdf]

ADHERENCE TO THE DASH DIET IN THE SPANISH POPULATION AND ITS ENVIRONMENTAL IMPACT: AN ECOLOGICAL STUDY

Supplementary Material 3, 4 and 5

Index

Supplementary Material S5 2

Supplementary Material S6 4

Supplementary Material S7 6

Supplementary Material S5

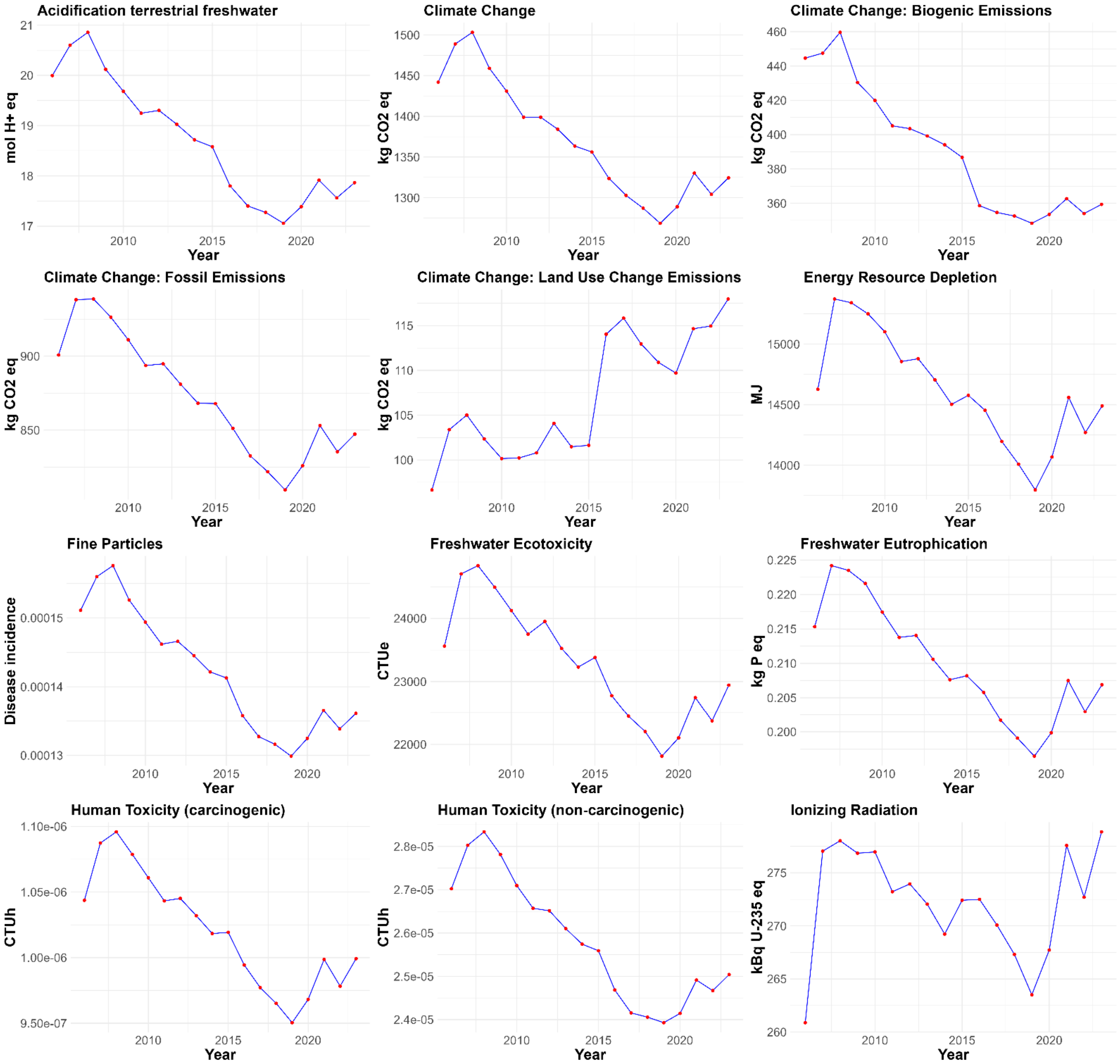

Supplementary Material S5: Environmental impact indicators trends

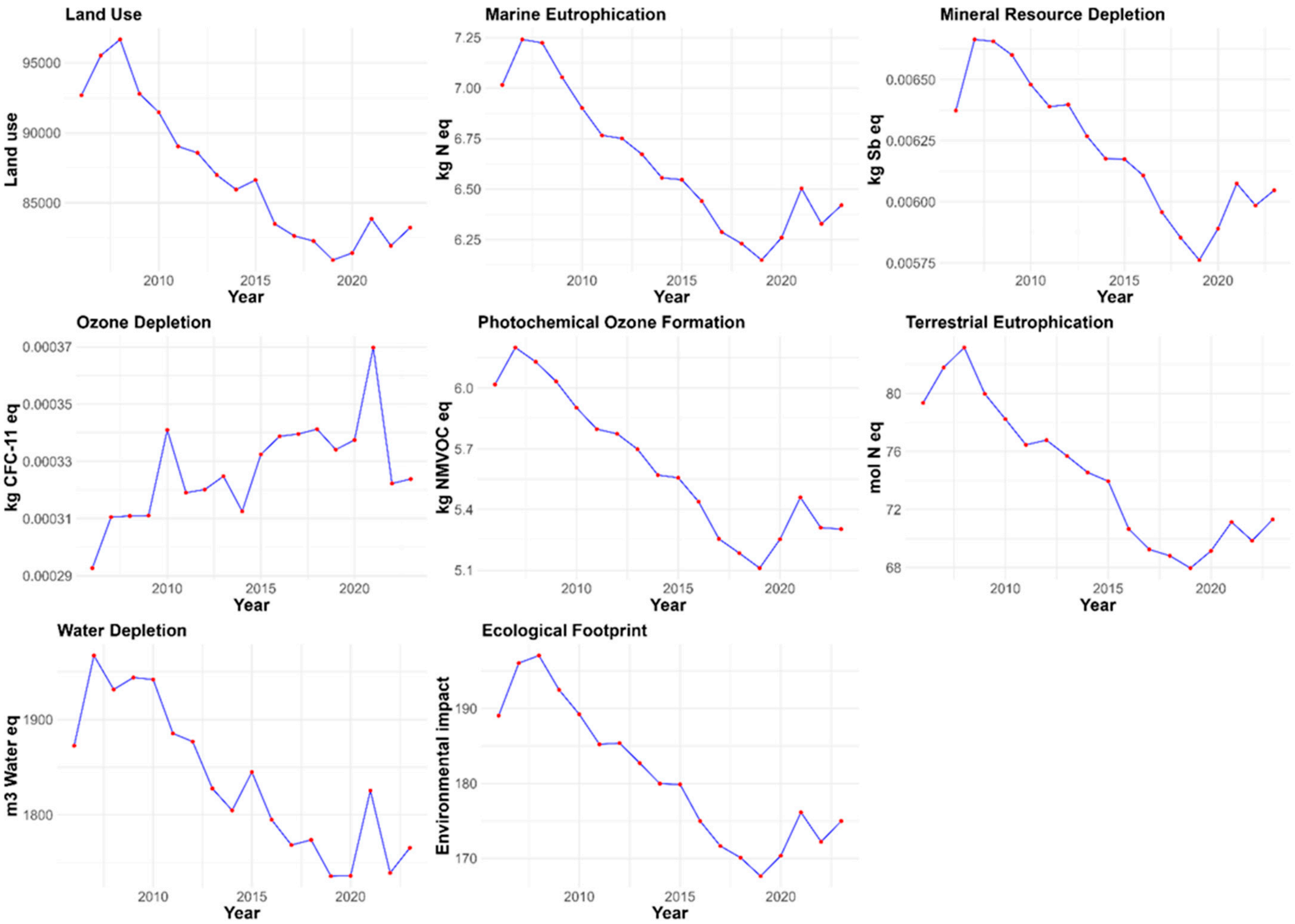

Supplementary Material S5 (cont.)

Supplementary Material S6

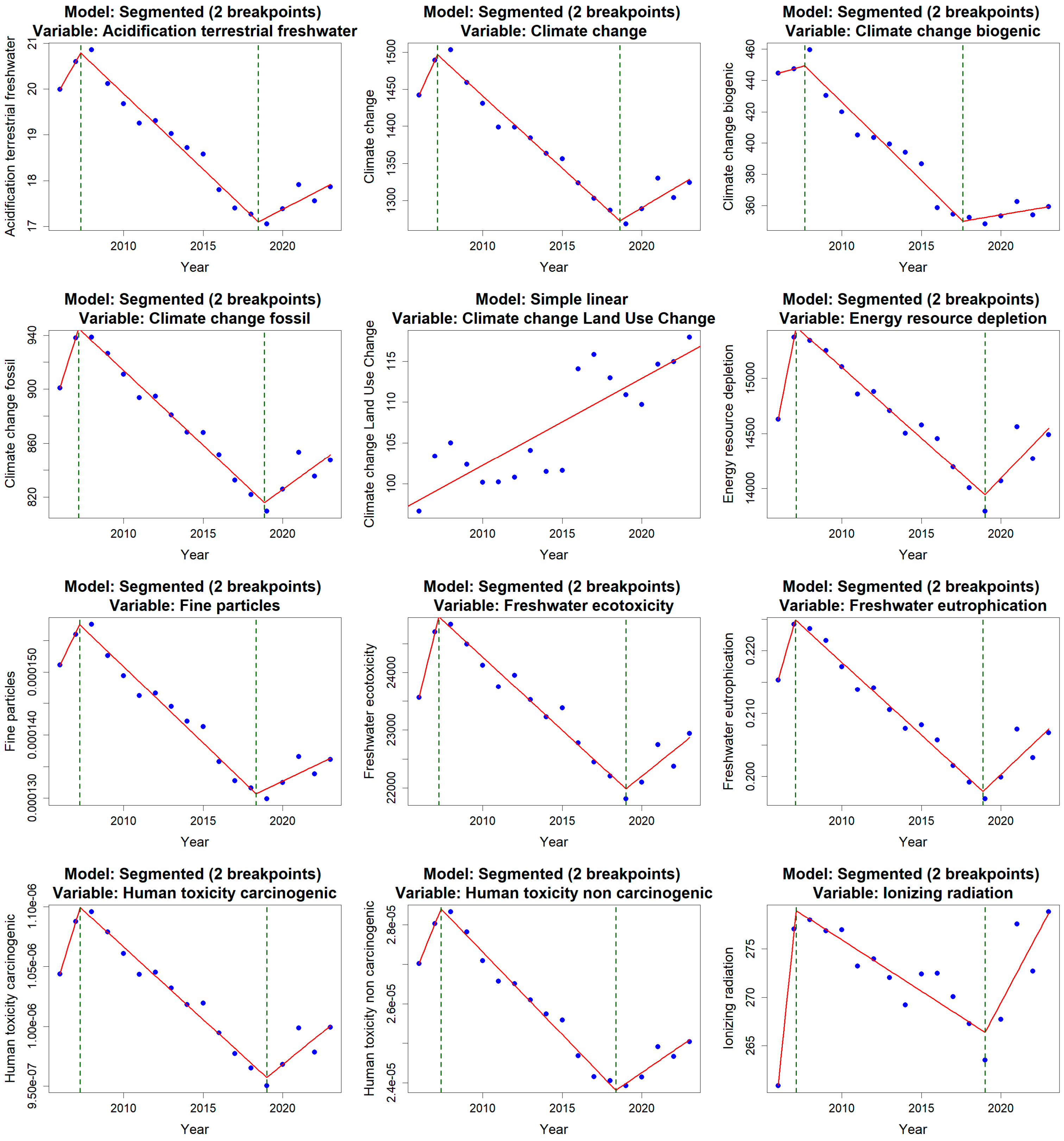

Supplementary Material S6: Graphs detailing each environmental impact and DASH trends

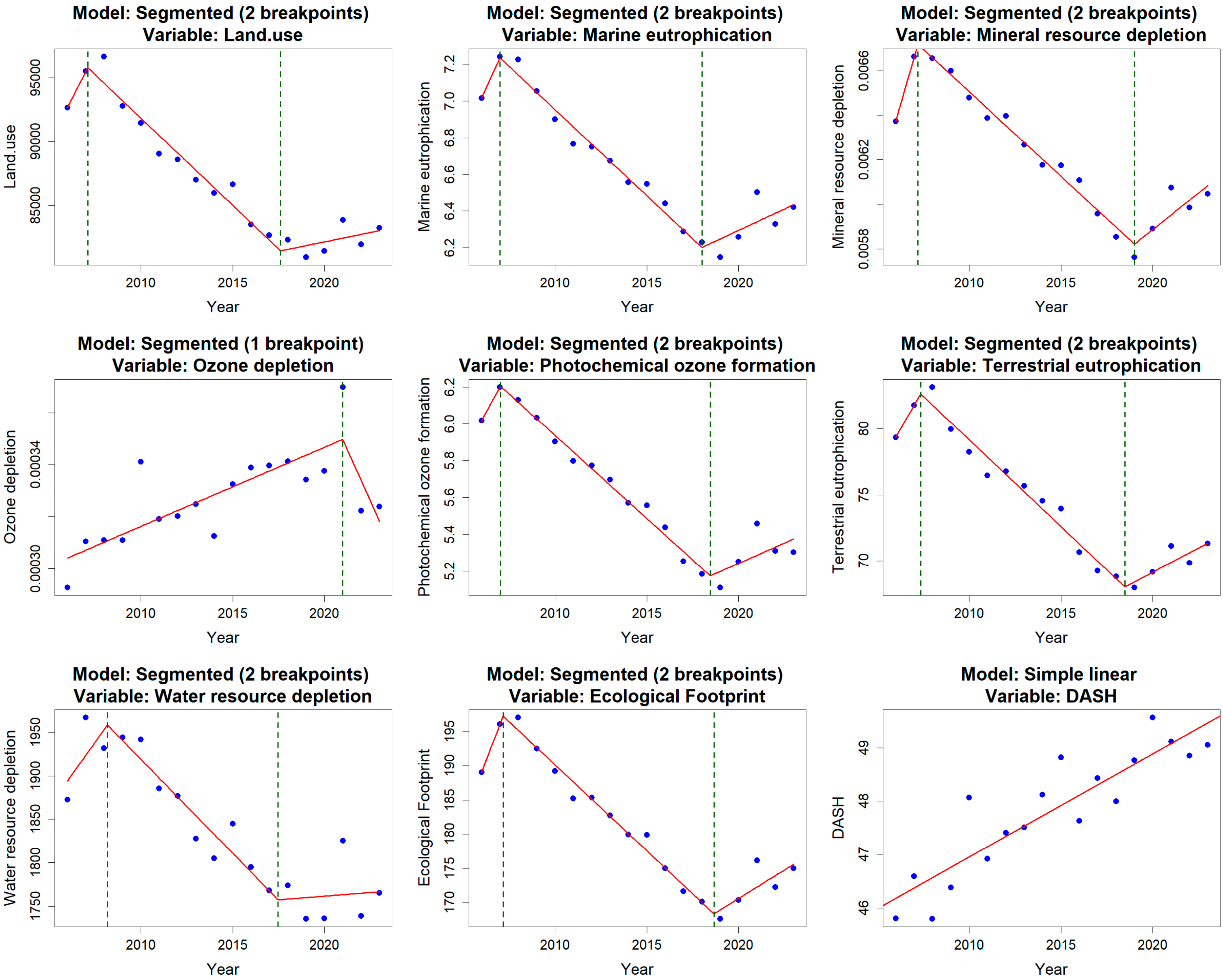

Supplementary Material S6 (cont.)

Supplementary Material S7

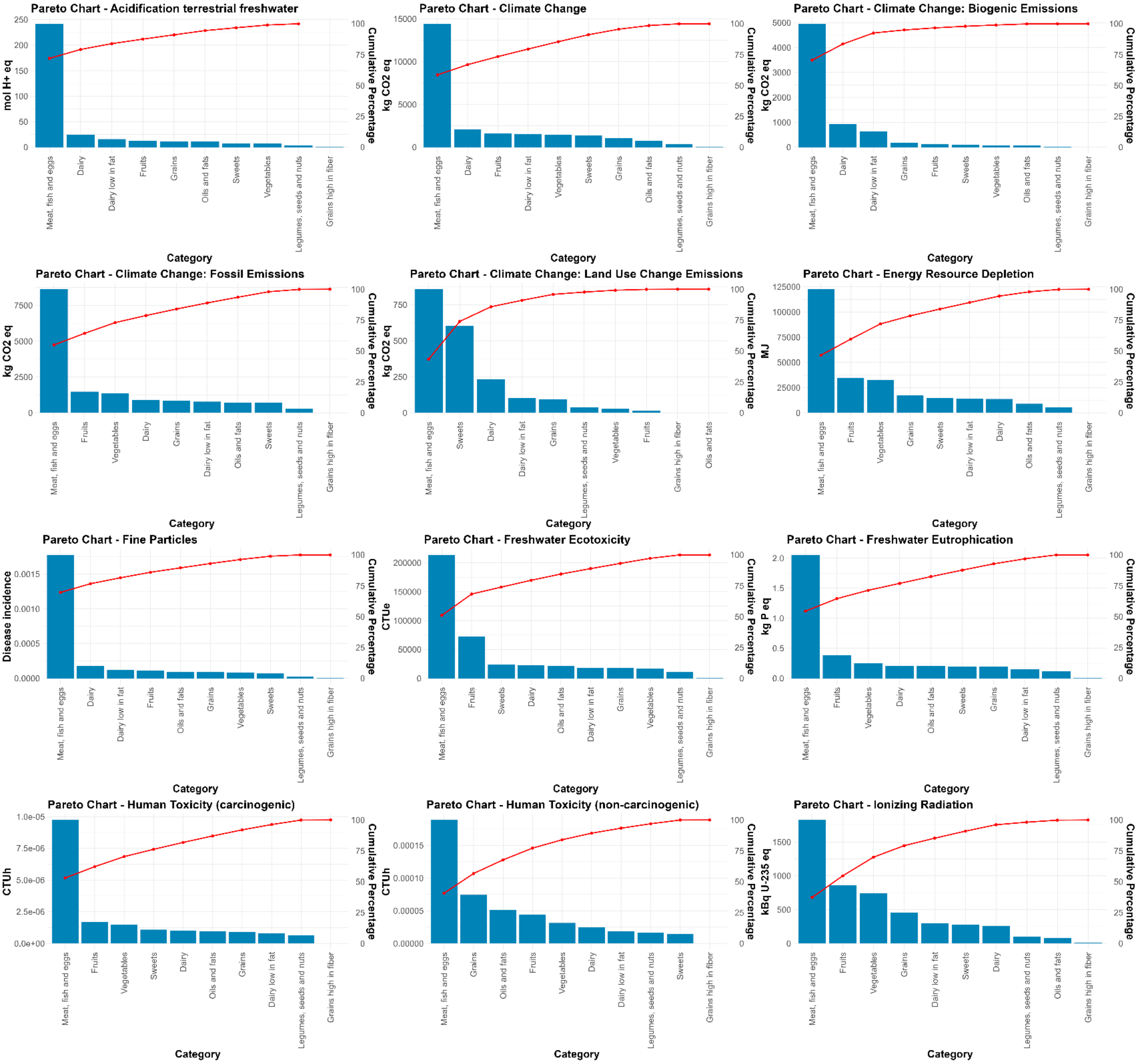

Supplementary Material S7: Pareto charts for all the environmental impact indexes

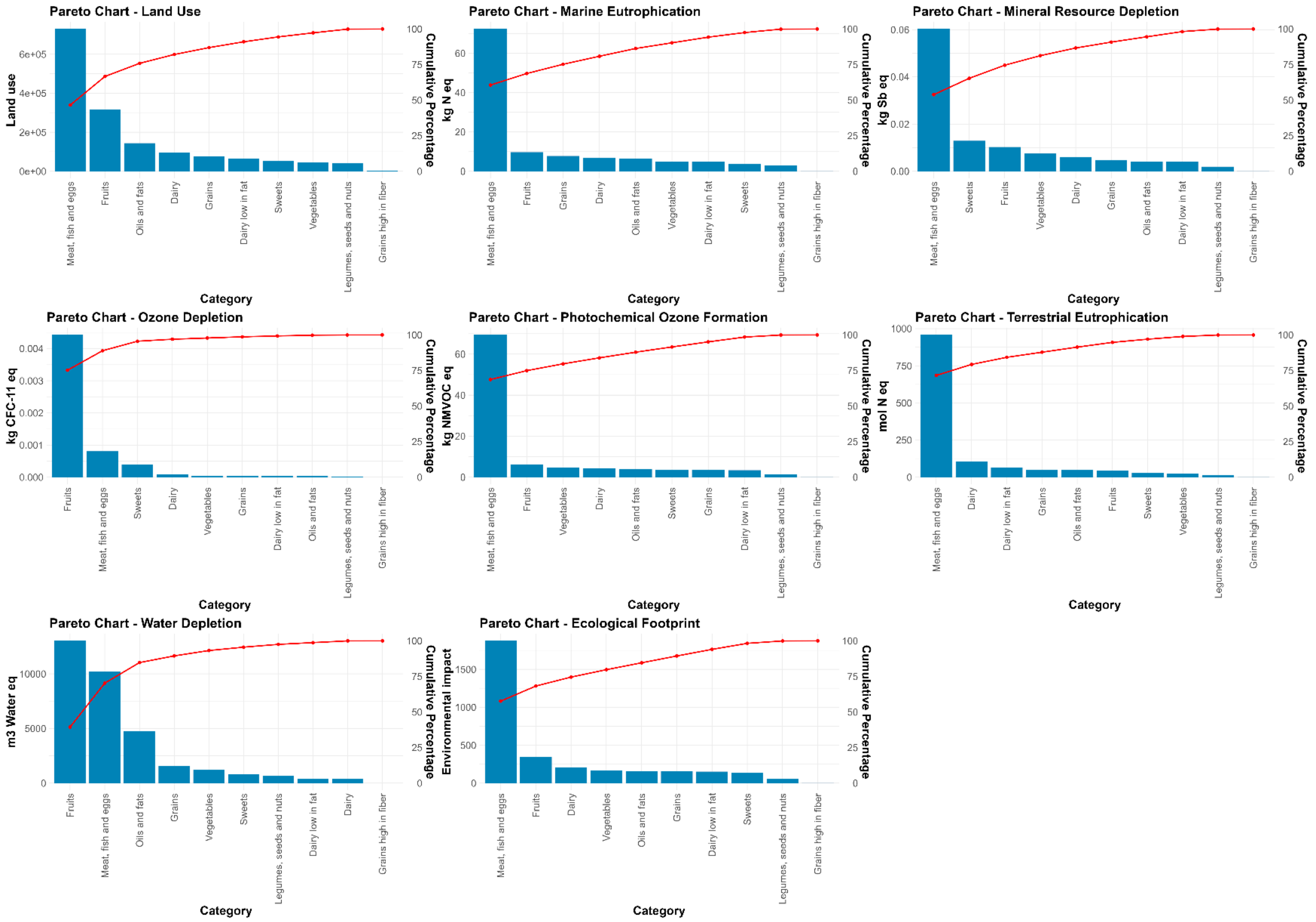

Supplementary Material S7 (cont.)
